# Erratum for “Pre‐clinical enteropathy in healthy soft‐coated wheaten terriers”

**DOI:** 10.1111/jvim.70034

**Published:** 2025-03-10

M. K. Tolbert, J. Darrow, L. Grubb, S. Fitzgerald, R. Bergee, J. Price, M. Mariano, M. Hong, C.‐H. Sung, T. Hill, J. M. Steiner and J. S. Suchodolski. Pre‐clinical enteropathy in healthy soft‐coated wheaten terriers. *Journal of Veterinary Internal Medicine*. 39, no. 2 (2025): e17293.

In the above mentioned article, Table 2, column “Healthy, non‐SCWT” n should equal 10.

